# Towards an optimized approach for de-labeling penicillin allergy: comparison between PEN-FAST and PEN-FAST+ in an Italian cohort

**DOI:** 10.3389/fphar.2026.1788547

**Published:** 2026-05-29

**Authors:** Giada Sambugaro, Vittoria Giagu, Maria Pina Barca, Andrea Giovanni Ledda, Giulia Costanzo, Stefano Del Giacco, Davide Firinu

**Affiliations:** Department of Public Health and Medical Science, University of Cagliari, Cagliari, Italy

**Keywords:** delabeling, delayed hypersensitivity, diagnostic score, drug hypersensitivity, PEN-FAST, PEN-FAST+, penicillin allergy

## Abstract

**Background:**

According to allergy work-up, fewer than 10% of adult and children who report a suspected penicillin allergy are actually allergic. The PEN-FAST score is currently validated for identifying patients at low-risk of penicillin allergy who benefit from a direct challenge test. The PEN-FAST+ has been developed with the aim of improving accuracy.

**Objectives:**

The aim of this study was to compare the performance of the PEN-FAST score and PEN-FAST+ score, with the aim of identifying the best clinical decision score to apply in daily practice to simplify and implement the de-labeling processes of penicillins allergy.

**Methods:**

Phase I: Retrospective evaluation of the PEN-FAST score and PEN-FAST+ score in patients with proven penicillin allergy. Phase II: Comparison of diagnostic performances of PEN-FAST and PEN-FAST+ scores.

**Results:**

288 patients were studied for suspected beta-lactams allergy. 245 had a reported history of penicillin allergy. A total of 32/245 (13%) patients were confirmed to be allergic to penicillin according to EAACI/ENDA definition. The PEN-FAST score showed limited discrimination capacity (AUC = 0.639 and NPV 93.75%). For immediate allergic penicillin, PEN-FAST misclassified 31% of patients (5/16) while PEN-FAST+ nobody. Both PEN-FAST and PEN-FAST+, misclassified 30% of patients (3/10) with delayed hypersensitivity. For unknown timing hypersensitivity reactions, PEN-FAST misclassified 33%% of patients (2/6) while PEN-FAST+ 67% (4/6). The AUC (0.810 vs. 0.639, p < 0.001) and NVP (96.59% vs. 93.75%), was significantly higher for PEN-FAST+ than PEN-FAST.

**Conclusion:**

Integrating PEN-FAST+ into diagnostic pathways could streamline patient selection for provocation tests, reduce false negatives, and optimize hospital resource use.

## Highlights

- Most reported penicillin allergies are incorrect, leading to unnecessary alternative antibiotic use and costs.
- PEN-FAST+ improves diagnostic accuracy in low-risk patients.
- Integrating PEN-FAST+ into practice can enhance safety and efficiency in penicillin allergy management

## 1 Introduction

Beta-lactam antibiotics are the most common cause of drug-related hypersensitivity reactions (DHRs), with a prevalence estimated to reach between 0.7% and 10% in the general population (Doña et al., 2024). According to allergy work-ups, fewer than 10% of adult and children who report a suspected penicillin allergy are actually allergic (Blumenthal et al., 2019; Romano et al., 2020). Labeling as penicillin-allergic is associated with increased morbidity and mortality, as alternative antibiotics may be less effective or expose patients to new adverse reactions. Additionally, alternative antibiotics can increase the risk of antibiotic resistance, and higher costs for the National Health Service (Castells et al., 2019; Blumenthal et al., 2020; MacFadden et al., 2016; Macy and Contreras, 2014). In the last decade, several strategies have been proposed to differentiate between allergic and non-allergic individuals and allow emergency use of penicillins. The current standard of care for diagnosing beta-lactam allergy includes epicutaneous tests and drug provocation tests. Unfortunately, the currently available serological tests for diagnosing beta-lactam allergy have significant limitations in sensitivity and specificity, therefore they have limited value in the diagnostic work-up and de-labeling (Romano et al., 2020). Risk stratification-based approaches can facilitate the evaluation of patients with suspected penicillin allergy, allowing for the selection of the most appropriate, safe, and rapid diagnostic iter (Barbaud et al., 2024). Validating the performance of clinical tools and standardized protocols is crucial for rapid delabeling, particularly in low-risk patients who could benefit from direct drug provocation test (DPT). Direct DPT has been shown to be safe and effective in low-risk penicillin allergy patients (Labella et al., 2025; Copaescu et al., 2023). To date, most studies focused on pediatric or non-European populations, but recent European evidence confirms the safety of a single-dose approach, supporting its use to further accelerate de-labeling (Labella et al., 2025). However, defining “low-risk” patients and standardizing risk stratification remain areas of ongoing debate (Sabato et al., 2021; Trubiano et al., 2020). PEN-FAST score allows the identification of low-risk patients for penicillin allergy who can benefit from direct DPT (Copaescu et al., 2023). The group of Castagna et al. recently present PEN-FAST+, a new clinical decision-making tool which increased the negative predictive value and the accuracy of PEN-FAST score (NPV 97% vs. 93% and AUC 85% vs.72%, p = 0.03), especially in patients with delayed hypersensitivity

Reactions (Castagna et al., 2023). Our study, performed at the Complex Program (PRGM-C) of Allergology and Clinical Immunology at Policlinico Universitario Duilio Casula in Monserrato (A.O.U. of Cagliari), wanted to evaluate the efficiency of the proposed new score, in our cohort of patients. We retrospectively compared the diagnostic performances of PEN-FAST and PEN-FAST+ scores in patients undergoing diagnostic procedures for suspected penicillin allergy, with the aim of identifying the best clinical decision score to apply in daily clinical practice to simplify and implement the delabeling processes of penicillin allergy.

## 2 Methods

The Institute’s Ethics Committee approved the study protocol (SIRTI - No.48 24 June 2025. All. 2.16). All collected data were anonymized before analysis. We report the study according to STARD (Standards for Reporting Diagnostic Accuracy Studies) guidelines (Bossuyt et al., 2015). The study was conducted in accordance with the Declaration of Helsinki.

### 2.1 Study participants

We enrolled 245 patients ≥ 14 years with a history of at least one adverse reaction to penicillins, who underwent a complete diagnostic work-up for suspected penicillin allergy. The lower age limit of 14 years reflects the local healthcare organisation, where patients below this age are referred to dedicated pediatric hospitals. The derivation cohort (from 2009 to 2025) consisted of 288 randomly selected patients admitted to the Allergology and Clinical Immunology Unit of the Cagliari University Hospital for suspected hypersensitivity reactions to beta-lactams. Only patients who underwent diagnostic workup to confirm penicillins allergy were included, while those having adverse reactions to another category of antibiotics were excluded. The diagnosis of penicillin allergy was confirmed or refuted based on validated protocols recommended by EAACI/ENDA, through the execution of *in-vivo* tests: skin tests (Skin Test ST) and/or drug provocation tests (Drug Provocation Test DPT) and *in-vitro* tests (specific IgE dosage - sIgE). The skin tests involved performing skin prick tests (SPT) followed by intradermal tests (ID) with benzylpenicilloate octa-L-lysine (BP-OL), benzylpenicillate (MDM), amoxicillin, ampicillin, clavulanic acid, and benzylpenicillin (DAP Penicillin Test kit - Diater; Madrid, Spain). The reading of SPT and ID was also performed at 48 and 72 h for those patients with a history of NIR or UR with a clinical phenotype presumably mediated by a type IV hypersensitivity. In cases of IR, alongside the skin tests, the serum levels of sIgE were also evaluated using ImmunoCAP (penicillin G, penicillin V, ampicillin, amoxicillin, and cefaclor), as well as the total IgE levels and serum tryptase. The patients who tested negative for the aforementioned tests underwent DPT.

### 2.2 Data collection

We reviewed the patients’ medical records and collected general anamnesis data regarding: age, gender, smoking history, and comorbidities. At the same time, data regarding the pharmacological history and detailed information about the suspected adverse reaction to penicillins were collected: the date of the suspected adverse reaction, the nature of the antibiotic molecule involved, the mode of onset, the type and severity of symptoms, and the evolution of symptoms with any need for anti-reactive therapy. Based on the timing of symptoms following drug administration, reactions were classified into immediate reactions (IR), non-immediate or delayed reactions (NIR), and reactions with unspecified timing (unknown-timing reactions, UR). IR, generally IgE-mediated, include reactions occurring within 1–6 h of beta-lactam intake (urticaria, angioedema, bronchospasm, or anaphylaxis up to anaphylactic shock). NIR include reactions occurring more than 1 h after drug exposure and are characterised by predominantly cutaneous manifestations, ranging from maculopapular exanthema to more severe hypersensitivity reactions. In cases where it was not possible to obtain precise information on the timing and symptoms of the adverse reaction, the reaction was classified as UR.

### 2.3 Study phases

The study was conducted in two phases. In the first phase of the study, we retrospectively applied the PEN-FAST score and the new PEN-FAST+ score to a cohort of patients undergoing the outpatient work-up procedure for penicillins allergy. In the second phase of the study, we compared the diagnostic performances of the two scores (PEN-FAST and PEN-FAST+) with the aim of identifying the best clinical decision score.

PEN-FAST is a clinical decision-making tool developed to identify patients at low risk of penicillin allergy. It was designed to simplify the diagnostic work-up by identifying low-risk patients who may proceed directly to DPT without prior skin testing, and is particularly useful in cases with a poorly suggestive clinical history. The name PEN-FAST is derived from the criteria used to calculate the score. Each criterion assigns a number of points, and the total score allows estimation of the patient’s risk level. “PEN” stands for penicillin, the antibiotic under evaluation. “F” (Five years or less since reaction): if the allergic reaction occurred within the past 5 years, 2 points are assigned. “A” (Anaphylaxis or angioedema) and “S” (Severe cutaneous adverse reaction): if severe symptoms such as anaphylaxis, angioedema, or severe cutaneous reactions occurred, 2 points are assigned. “T” (Treatment required or unknown): if the reaction required treatment or if details are unknown, 1 point is added. A score of <3 points indicates a low risk of penicillin allergy, with a high negative predictive value (NPV), and the patient may therefore proceed directly to a DPT with the culprit drug. A score of ≥3 points indicates a higher risk, and skin tests or further evaluation are recommended before administering penicillin (Trubiano et al., 2020; Copaescu et al., 2023).

PEN-FAST+ is an improved version of the PEN-FAST clinical score, designed to enhance the effectiveness of risk stratification and to address some of the limitations of the original tool, particularly for non-immediate reactions (e.g., maculopapular exanthema) (Castagna et al., 2023). Unlike its predecessor, in PEN-FAST+ a reaction occurring within the past 5 years assigns only 1 point; the same applies to severe reactions (anaphylaxis, angioedema, or SCAR). The point previously assigned for treatment requirement is removed, and two additional criteria are introduced: a skin rash lasting more than 7 days assigns 2 points; an immediate reaction (IR) occurring within 1 h assigns an additional 2 points. In this context, IR also includes symptoms such as localised pruritus or a sensation of warmth (palmoplantar or on the scalp), which may represent early precursors of a significant allergic reaction. A score of <2 points indicates a low risk of penicillin allergy, with a high probability that the patient is not truly allergic. In these cases, a DPT may be performed directly without preliminary testing. A score of ≥2 points indicates a higher risk, and it is recommended to proceed according to standardised protocols (ST followed by DPT).

### 2.4 Statistical analysis

All collected data were anonymized before analysis. The statistical analyses were performed using SPSS version 29 and Jamovi 2.6.44 programs. Continuous variables are reported as mean ± standard deviation (SD) or median (interquartile range), while categorical data are reported as counts and percentages. The PEN-FAST and PEN-FAST+ scores were compared with the results of validated tests used to confirm an allergy to penicillins (positive ST or DPT). Sensitivity, specificity, negative predictive value (NPV), positive predictive value (PPV), accuracy, and the area under the receiver operating characteristic curve (ROC) were calculated to evaluate the overall diagnostic performance. Sensitivity was defined as the probability that an allergic patient was classified as at risk of allergy (PEN-FAST score ≥ 3 and PEN-FAST+ ≥ 2). Specificity was defined as the probability that a non-allergic patient was classified as low risk for allergy, having a PEN-FAST score < 3 or PEN-FAST+ < 2. The NPV was defined as the probability that a patient classified as low risk for allergy was non-allergic, and the PPV as the probability that a patient classified as high risk for allergy was truly allergic to penicillin. A two-tailed p-value <0.05 was considered statistically significant. The area under the ROC curve of the two scores was compared using the DeLong test.

## 3 Results

### 3.1 Demographic data, comorbidities and global assessment of adverse reactions

**FIGURE 1:**
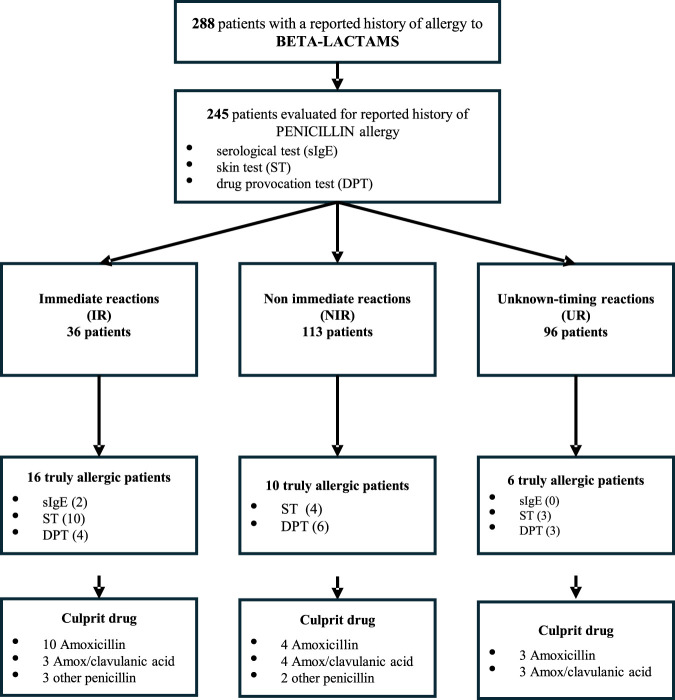
Patients undergoing allergological evaluation for a history of penicillin allergy.

A total of 288 patients underwent allergological evaluation for a history of beta-lactam (BL) allergy. The median age of the patients was 46.3 years and 77.7% were females (N = 224). The 12.15% (35/288) were smokers or ex-smokers and the 46.88% (135/288) had at least one comorbidity: Th2-related diseases (atopic dermatitis, allergic rhinitis, eosinophilic asthma, food allergy, eosinophilic esophagitis) in 53.33% (72/135), cardiovascular diseases in 23.70% (32/135), autoimmunity in 28.89% (39/135), pulmonary diseases in 15.56% (21/135), kidney disease 0.74% (1/135). 245 met the inclusion criteria and were included in the subsequent analyses. At the end of all the procedures carried out in the diagnostic work-up of patients according to EAACI/ENDA guidelines, only 12.85% (37/288) of the examined cases confirmed an allergic hypersensitivity to BL. Specifically, 32/245 confirmations of allergy to penicillins (13.06%) and 5/43 confirmations of allergy to cephalosporins (11.63%). Of the 245 suspected adverse reactions to penicillins, 32.24% (N = 79) were reported to amoxicillin, 51.43% (N = 126) to amoxicillin and clavulanic acid, and 16.33% (N = 40) to other penicillins. Penicillin allergy was confirmed in 32/245 patients (13.06%), of whom 50% (N = 16) reported an immediate adverse reaction (IR), 31.25% (N = 10) a non-immediate adverse reaction (NIR), and 18.75% (N = 6) an unknown-timing reaction (UR). In the 16 patients with a history of IR, the diagnosis of immediate allergic hypersensitivity was confirmed in 2 patients with positive serological tests, 10 patients with skin tests and in 4 patients during the performance of the DPT. In the 10 patients with a history of NIR, the diagnosis of delayed-type allergic hypersensitivity was confirmed in 4 patients with delayed reading skin tests (LR) and in 6 patients with DPT. In the 6 patients with a reaction with imprecise timing, 3 patients tested positive on the skin tests and 3 to DPT (Table 1; Figure 1). None of the patients that underwent skin tests experienced immediate systemic adverse reactions. In none of the drug challenges a systemic or severe immediate adverse reaction occurred. Thus, about 87% of the patients (213/245) evaluated in our study were not truly allergic to penicillin (Supplementary Table).

**TABLE 1:** Cohort characteristics and reaction details.

Characteristic	N (%)
Overall cohort of beta-lactam reactions (N = 288)	
Median age, years	46.3
Female	224 (77.7%)
Smokers or ex-smokers	35 (12.15%)
At least one comorbidity	135 (46.88%)
* Th2-related diseases*	72 (53.33%)
* Cardiovascular diseases*	32 (23.70%)
* Autoimmunity*	39 (28.89%)
* Pulmonary diseases*	21 (15.56%)
* Kidney disease*	1 (0.74%)
Reaction to penicillins	245 (85.07%)
Reaction to cephalosporins	43 (14.93%)
Penicillin allergy workup (N = 245)	
Amoxicillin	79 (32.24%)
Amoxicillin/clavulanic acid	126 (51.43%)
Other penicillins	40 (16.33%)
Confirmed penicillin allergy	32 (13.06%)
* *Immediate reaction (IR)	16 (50%)
* *Confirmed by serology	2
* *Confirmed by skin tests	10
* *Confirmed by DPT	4
* *Non-immediate reaction (NIR)	10 (31.25%)
* *Confirmed by delayed skin tests	4
* *Confirmed by DPT	6
* *Unknown-timing reaction (UR)	6 (18.75%)
* *Confirmed by skin tests	3
* *Confirmed by DPT	3

IR: immediate reaction; NIR: non-immediate reaction; UR: unknown-timing reaction; DPT: drug provocation test.

### 3.2 Retrospective performance of the PEN-FAST score and PEN-FAST+ scores

Among the 32 patients with confirmed penicillin allergy, 22/32 (68.75%) were correctly identified by a PEN-FAST score ≥3 points, while the remaining 10/32 (31.25%) were misclassified (PEN-FAST <3). Among the same 32 patients, 25/32 (78.12%) were correctly identified by a PEN-FAST+ score ≥2 points, while only 7/32 (21.88%) were misclassified (PEN-FAST+ <2) despite positive skin tests or drug provocation tests (supplementary table). The full diagnostic performance of both scores is shown in Table 2.

**TABLE 2:** Diagnostic Performance of PEN-FAST and PEN-FAST+ score.

Diagnostic performance	PEN-FAST	PEN-FAST+
Sensitivity	68.75% (49.99%–83.88%)	78.12% (60.03%–90.72%)
Specificity	58.59% (52.29%–64.69%)	77.34% (71.72%–82.32%)
Positive likelihood ratio	1.660 (1.261–2.187)	3.448 (2.577–4.614)
Negative likelihood ratio	0.533 (0.316–0.901)	0.283 (0.146–0.546)
Prevalence	11.11% (7.73%–15.32%)	11.11% (7.73%–15.32%)
Positive predictive value	17.19% (13.61%–21.47%)	30.12% (24.36%–36.58%)
Negative predictive value	93.75% (89.88%–96.20%)	96.59% (93.61%–98.20%)
Accuracy	59.72% (53.81%–65.43%)	77.43% (72.16%–82.13%)

Values expressed as Result (Lower 95% CI – Upper 95% CI). PEN-FAST: low risk <3; high risk ≥3. PEN-FAST+: low risk <2; high risk ≥2.

### 3.3 Comparison of the diagnostic performance of PEN-FAST score and PEN-FAST+ score

**FIGURE 2:**
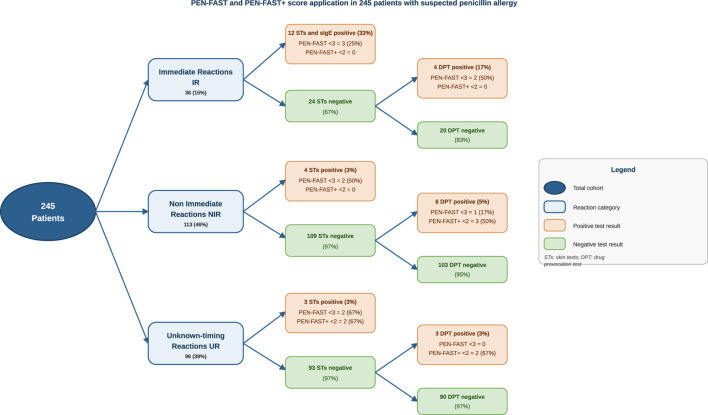
Characteristics of reactions observed in the reported cohort of patients, and results of the application of the PEN-FAST and PENFAST+ scores.

**FIGURE 3:**
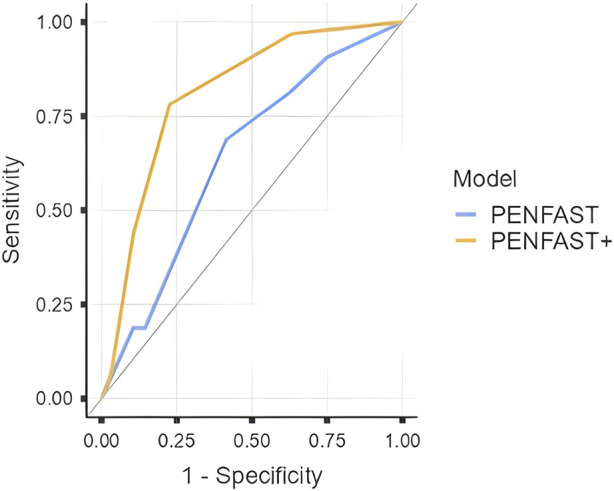
Estimated AUC’s: 0.639 vs. 0.810; p-value < 0.001 (Delong test).

The PEN-FAST score showed limitations in predicting the recurrence of immediate-type hypersensitivity reactions and non-severe delayed cutaneous reactions. After examining the clinical records of patients misclassified with PEN-FAST, we reapplied the PEN-FAST+ score and compared the diagnostic performances of the two scores. The characteristics of patient reactions, the allergological analysis, and the results are shown in Figure 2. PEN-FAST misclassified 31% (5/16) of IR patients, while PEN-FAST+ misclassified none. Both scores misclassified 30% (3/10) of NIR patients. For UR, PEN-FAST misclassified 33% (2/6) and PEN-FAST+ 67% (4/6). Overall, PEN-FAST+ correctly identified 78.12% (25/32) of truly allergic patients compared to 68.75% (22/32) for PEN-FAST. The AUC was significantly higher for PEN-FAST+ than PEN-FAST (81% vs. 63%, p < 0.001, DeLong test) (Figure 3).

## 4 Discussion

In this study, 288 patients with a positive history of adverse reactions to beta-lactams were studied through a complete diagnostic process; 245 patients met the inclusion criteria (history of adverse reactions to penicillins). Our results demonstrated that PEN-FAST+ outperforms PEN-FAST in risk stratification of penicillin allergy, showing significantly higher AUC (0.810 vs. 0.639, p < 0.001) and NPV (96.59% vs. 93.75%), particularly in the classification of immediate reactions. These findings suggest that the integration of PEN-FAST into clinical pathways could improve patient selection for provocation tests, reduce false negatives, and optimize healthcare resources.

The DHRs mainly involved amoxicillin and amoxicillin/clavulanic acid, a fact consistent with the widespread use of these drugs nationally over the past decade. At the end of the complete diagnostic process according to EAACI/ENDA guidelines, 32 patients were truly allergic. The skin tests and the challengetests with the culprit drug or alternative showed a high level of safety, no patient that underwent to skin tests experienced immediate systemic adverse reactions. Among the challenge tests performed, in no case were there immediate systemic or severe adverse reactions.

Mis-labeling for penicillin allergies remains a current and increasingly widespread issue. Currently, the Allergy departments in an outpatient setting are overwhelmed by evaluations for “suspected penicillin allergy”, yet most patients are incorrectly diagnosed with an allergy. This may lead to the use of alternative antibiotics that may be less effective or safe, contribute to increased antibiotic resistance, longer hospital stays and higher healthcare costs. This highlights the need to validate protocols and clinical support tools for rapid de-labeling, particularly in centers where limited resources, staffing, or space make it difficult to carry out currently approved de-labeling protocols (Ghiordanescu et al., 2024). One of the first clinical tools approved for risk stratification was the PEN-FAST (Trubiano et al., 2020; Copaescu et al., 2023; Piotin et al., 2022; Su et al., 2023; Tran et al., 2025; Hanniet et al., 2024). However, Castagna et al. have highlighted how relying on PEN-FAST can lead to misclassifying a significant portion of patients, particularly those with a history of delayed reaction, among the most frequently encountered DHRs to penicillins in clinical practice (Castagna et al., 2023). In our study, the retrospective application of PEN-FAST showed an NPV of 93.75% and an AUC of 0.639. PEN-FAST+ in the same cohort resulted in a significantly higher NPV of 96.59% and AUC of 0.810 (p < 0.001), confirming the findings of the original French study (Castagna et al., 2023). PEN-FAST+ showed a clear advantage over PEN-FAST in the classification of immediate reactions, correctly identifying all 16 IR patients, while PEN-FAST misclassified 31% (5/16). For non-immediate reactions, both scores performed similarly, each misclassifying 30% (3/10) of NIR patients. For reactions with undetermined timing, PEN-FAST+ showed an inferior performance, misclassifying 67% (4/6) versus 33% (2/6) for PEN-FAST. We believe this is largely attributable to a recall bias: given that most reactions had occurred more than 5 years before assessment, many patients were unable to accurately recall the timing and clinical features of their reaction, leading to a higher proportion of UR classifications and impairing the performance of any timing-dependent scoring tool. This reflects a common scenario in daily practice, and underscores the pivotal role of the physician who first encounters a suspected hypersensitivity reaction. Thorough documentation of timing, clinical features, and severity at the time of the reaction would substantially reduce the proportion of UR classifications and allow downstream allergological risk stratification to be performed on a more solid clinical basis. Promoting this awareness among primary care and emergency medicine practitioners represents an important, and currently underappreciated, component of effective penicillin allergy management. Regarding the clinical phenotypes of the confirmed allergic patients, the predominant manifestations were urticaria and maculopapular exanthema. Anaphylaxis cases were present but represented a minority of the cohort (n = 2), which mitigates the concern that the superior performance of PEN-FAST+ might be driven by an over-representation of high-severity reactions. Importantly, no cases of SCAR, such as Stevens-Johnson syndrome or toxic epidermal necrolysis, were included. We agree with the current literature that SCAR represents an absolute contraindication to the use of risk stratification de-labeling tools (Castagna et al., 2023; Ghiordanescu et al., 2024). Compared to other scoring systems reported in the literature, PEN-FAST+ demonstrates competitive performance. The PALACE trial validated PEN-FAST showing strong NPV in low-risk patients selected for direct oral challenge (Copaescu et al., 2023). Ghiordanescu et al. compared four penicillin allergy prediction strategies in a large cohort, highlighting the variable performance of existing tools across different clinical settings (Ghiordanescu et al., 2024). Hanniet et al. validated PEN-FAST in a French cohort, reporting results broadly consistent with our own (Hanniet et al., 2024). The work of Castagna et al. introducing PEN-FAST+ showed an NPV of 97% and AUC of 85% in their derivation cohort (Castagna et al., 2023), figures slightly higher than those observed here (NPV 96.59%, AUC 81%), which may reflect differences in population characteristics, reaction phenotype distribution, or centre-specific diagnostic protocols. Although the relatively small sample size (n = 245), the monocentric design, and the limited number of patients in the NIR and UR subgroups (10 and 6, respectively) somewhat limit the external generalizability of our results, the cohort was representative of real-life referrals to allergy outpatient clinics. Future prospective, multicentre studies enrolling patients across all reaction phenotypes, and ideally at earlier time points after the index reaction, are needed to confirm these findings, including in pediatric populations, in whom validated data are currently lacking. Overall, our data support the integration of PEN-FAST+ into allergy workups as a step toward safer, evidence-based de-labelling strategies and improved antimicrobial stewardship (Trubiano et al., 2020; Copaescu et al., 2023; Castagna et al., 2023; Ghiordanescu et al., 2024; Piotin et al., 2022; Su et al., 2023; Tran et al., 2025; Hanniet et al., 2024).

## 5 Conclusions

In conclusion, our study highlights the importance of a standardized and accurate diagnostic approach for beta-lactam hypersensitivity, particularly to penicillins. Only a minority of patients with a history of hypersensitivity were confirmed to be truly allergic, underscoring the problem of overdiagnosis and the need for effective de-labeling strategies. Allergen testing, including skin and challenge tests, proved to be safe, with a very low rate of adverse reactions. While PEN-FAST remains a useful risk stratification tool, it has limitations in correctly identifying patients with immediate and undetermined-timing reactions. The PEN-FAST + score demonstrated higher diagnostic accuracy and NPV, suggesting that its integration into clinical pathways could improve patient selection for provocation tests, reduce false negatives, and optimize healthcare resources. Overall, more precise tools like PEN-FAST+ may enhance the safety and efficiency of penicillin allergy management, particularly in settings with limited resources. Our findings further support the integration of PEN-FAST+ into allergy workups as a step toward safer, evidence-based de-labeling strategies and improved antimicrobial stewardship. Future prospective, multicentre studies, ideally enrolling patients at earlier time points after the index reaction and including pediatric populations, are warranted to confirm these findings and support the broader implementation of PEN-FAST+ in clinical practice.

## Data Availability

The raw data supporting the conclusions of this article will be made available by the authors, without undue reservation.
