# Supplementary material for: Towards an optimized approach for de-labeling penicillin allergy: comparison between PEN-FAST and PEN-FAST+ in an Italian cohort
Source: Front Pharmacol. 2026 May 29;17:1788547. doi: 10.3389/fphar.2026.1788547 (PMC13260553; doi:10.3389/fphar.2026.1788547)
Supplement: Supplementary file 1 [file Table1.docx]

**Supplementary Table – Clinical Characteristics and Scores of All 32 Patients :** *IR = Immediate Reaction; NIR = Non-Immediate Reaction; UR = Undetermined Reaction; DAP = Drug Allergy Panel; Amox = Amoxicillin; Aug = Augmentin PEN-FAST: low risk <3; high risk ≥3. PEN-FAST+: low risk <2; high risk ≥2*

| **Patient #** | **Sex** | **Age (y)** | **Culprit Drug** | **Reaction Timing** | **Reaction Type** | **Clinical Symptoms** | **Therapy Required** | **Adrenaline Required** | **Allergy Confirmed by** | **PEN- FAST** | **PEN- FAST+** |
| --- | --- | --- | --- | --- | --- | --- | --- | --- | --- | --- | --- |
| 1 | M | 64 | Amoxi/clav | <5y | IR | Urticaria | No | No | Skin tests (DAP) | 2 | 3 |
| 2 | F | 69 | Amox | >5y | IR | Anaphylactic shock | Yes | Yes | Skin tests (DAP) | 3 | 3 |
| 3 | F | 51 | Amoxi/clav | <5y | NIR | Delayed urticaria | yes | No | Provocation test | 3 | 1 |
| 4 | F | 77 | Amox | >5y | UR | Skin rash  lasting >7 days | yes | No | Skin tests (DAP) | 1 | 2 |
| 5 | F | 74 | Other | <5y | IR | Urticaria | yes | No | Skin tests (DAP) | 3 | 3 |
| 6 | F | 62 | Amoxi/clav | >5y | IR | Urticaria | yes | No | Skin tests (DAP) | 1 | 2 |
| 7 | F | 41 | Other | <5y | NIR | Skin rash  lasting >7 days | yes | No | Skin tests (DAP) | 3 | 3 |
| 8 | F | 48 | Amox | >5y | NIR | Skin rash  lasting >7 days | yes | No | Skin tests (DAP) | 1 | 2 |
| 9 | F | 67 | Amoxi/clav | >5y | NIR | Skin rash  lasting >7 days | Yes | No | Skin tests (DAP) | 1 | 2 |
| 10 | F | 86 | Amox | <5y | IR | Urticaria | Yes | No | Skin tests (DAP) | 3 | 3 |
| 11 | F | 59 | Other | >5y | IR | Urticaria | No | No | Provocation test | 0 | 2 |
| 12 | F | 18 | Other | <5y | IR | Skin rash  lasting <7 days | No | No | Skin tests (DAP) | 2 | 2 |
| 13 | F | 30 | Amox | >5y | UR | Angioedema | No | No | Skin tests (DAP) | 2 | 1 |
| 14 | F | 24 | Amox | <5y | IR | Urticaria | Yes | No | Skin tests (DAP) | 3 | 3 |
| 15 | F | 37 | Amox | <5y | IR | Urticaria | Yes | No | Skin tests (DAP) | 3 | 3 |
| 16 | F | 64 | Other | <5y | NIR | Delayed Urticaria | No | No | Provocation test | 2 | 1 |
| 17 | F | 40 | Amox | <5y | IR | Urticaria | Yes | No | Provocation test | 3 | 3 |
| 18 | F | 54 | Other | <5y | IR | Aspecific (pruritus) | Yes | No | Provocation test | 3 | 3 |
| 19 | F | 56 | Other | <5y | IR | Urticaria | Yes | No | Serology | 3 | 3 |
| 20 | F | 42 | Amox | <5y | NIR | Delayed urticaria | Yes | No | Provocation test | 3 | 1 |
| 21 | F | 59 | Amox | >5y | IR | Aspecific (pruritus) | No | No | Provocation test | 0 | 2 |
| 22 | F | 16 | Amox and Amoxi/clav | <5y | NIR | Skin rash  lasting >7 days | Yes | No | Provocation test | 3 | 3 |
| 23 | F | 51 | Amox and Amoxi/clav | <5y | NIR | Skin rash  lasting >7 days | Yes | No | Provocation test | 3 | 3 |
| 24 | F | 59 | Amox and Amoxi/clav | <5y | UR | Angioedema | Yes | No | Provocation test | 5 | 2 |
| 25 | F | 50 | Amoxi/clav | <5y | NIR | Skin rash lasting<7 days + Angioedema | Yes | No | Provocation test | 5 | 2 |
| 26 | F | 44 | Amox | >5y | UR | Urticaria + Angioedema | Yes | No | Provocation test | 3 | 1 |
| 27 | F | 55 | Amoxi/clav | >5y | UR | Urticaria + Angioedema | Yes | No | Provocation test | 3 | 1 |
| 28 | F | 62 | Amoxi/clav | >5y | IR | Bronchospasm | Yes | No | Skin tests (DAP) | 3 | 3 |
| 29 | F | 61 | Amoxi/clav | <5y | UR | Urticaria | Yes | No | Skin tests (DAP) | 3 | 1 |
| 30 | F | 30 | Amoxi/clav | >5y | IR | Angioedema | Yes | No | Skin tests (DAP) | 3 | 3 |
| 31 | F | 47 | Amoxi/clav | <5y | IR | Anaphylaxis | Yes | No | Serology | 5 | 4 |
| 32 | F | 40 | Amox/clav | <5y | NIR | Skin rash  lasting >7 days | Yes | No | Skin tests (DAP) | 3 | 2 |
